# Retrograde intramedullary nailing for the treatment of femoral medial condyle fracture nonunion

**DOI:** 10.1007/s11751-015-0215-5

**Published:** 2015-03-13

**Authors:** Takahiro Niikura, Sang Yang Lee, Yoshitada Sakai, Kotaro Nishida, Ryosuke Kuroda, Masahiro Kurosaka

**Affiliations:** Department of Orthopaedic Surgery, Kobe University Graduate School of Medicine, 7-5-1 Kusunoki-cho, Chuo-ku, Kobe, 650-0017 Japan

**Keywords:** Femur, Medial condyle fracture, Nonunion, Retrograde intramedullary nail

## Abstract

An unicondylar fracture of the femur is uncommon and of the medial condyle more so. Open reduction and internal fixation of these fractures is most commonly performed with screws or plate and screws. Secure bone fixation is compromised by osteoporosis in elderly patients; additional measures may be required. We report the case of an elderly osteoporotic patient with a medial condyle fracture nonunion treated successfully through retrograde intramedullary nailing. A 78-year-old osteoporotic woman suffered medial condyle fracture of the femur 9 months before visiting our hospital. She had been treated conservatively, and the fracture demonstrated a complete nonunion with gross instability. The edge fragments appeared sclerotic, and the nonunion site was accompanied by a bony defect. Although fixation by a plate and screw is the standard method for the treatment of such fracture, we judged that stability would be difficult to achieve with this method due to the accompanying bony defect and osteoporosis. Thus, we performed open reduction and fixation by retrograde intramedullary nailing with the use of “condyle screw and nut” system, followed by bone grafting. Bony union was successfully obtained. The stability and range of motion of the knee were recovered, and the patient regained the ability to walk. We suggest the unique application of retrograde intramedullary nailing with condyle screw and nut for the treatment of specific, complex cases of femoral medial condyle fracture.

## Introduction

Unicondylar fractures of the femur are uncommon and accounts for 0.65 % of all femoral fractures [1]. In addition, a fracture of the medial condyle is rarer with the lateral condyle involved three times as often as the medial condyle in reports of unicondylar fractures [1]. Nonunion of these unicondylar fractures is rarely met. We report the case of an elderly patient with a pseudoarthrosis of a medial femoral condyle fracture which had been treated nonoperatively in the first instance and led to nonunion with gross instability and evidence of a bony defect between the fragments.

Open reduction and internal fixation is recommended for femoral unicondylar fractures to reduce the possibility of posttraumatic arthritis, joint contracture, and knee instability. This is achieved with standard surgical techniques such as screw fixation or plate/screw fixation [2–5]. Osteoporosis in elderly patients compromises secure bone fixation by screw and plate fixation [5] and warrants additional measures to obtain stable fixation.

## Case report

**Fig. 1:**
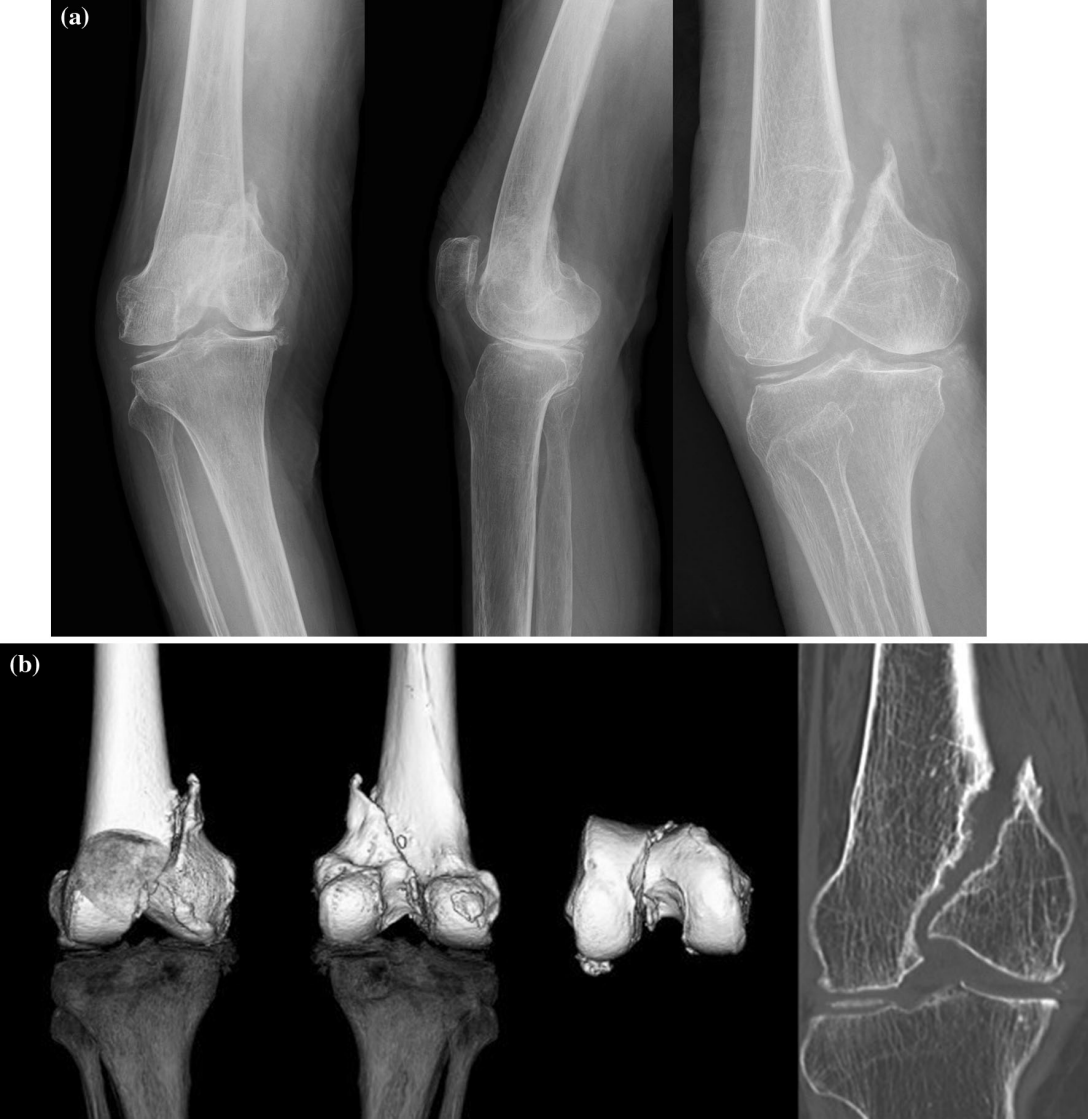
**a** Radiographs demonstrating medial condyle fracture nonunion of the femur. **b** computed tomography demonstrating the details of the fracture nonunion

A 78-year-old woman slipped on a slope and suffered a medial condyle fracture of the femur (33-B2 by AO/OTA classification) 9 months prior to the presenting at our hospital. She had been treated conservatively, and the fracture was diagnosed to have developed into a pseudoarthrosis with gross instability. The fracture edges were sclerotic, and the pseudoarthrosis showed evidence of a bony defect (Fig. 1). The patient’s chief complaint was instability of the knee joint followed by gait disturbance. She could walk only short distances with a knee support and a cane. The gait disturbance showed a marked lateral thrust. The range of motion of the knee was noted as 10°–115°. There were comorbidities: the patient had diabetes mellitus and osteoporosis. Her HbA1c was measured at 8.3 %, and her bone mineral density of the lumbar spine was 63 % of the young adult mean. No medication had been prescribed previously for treatment of osteoporosis.

**Fig. 2:**
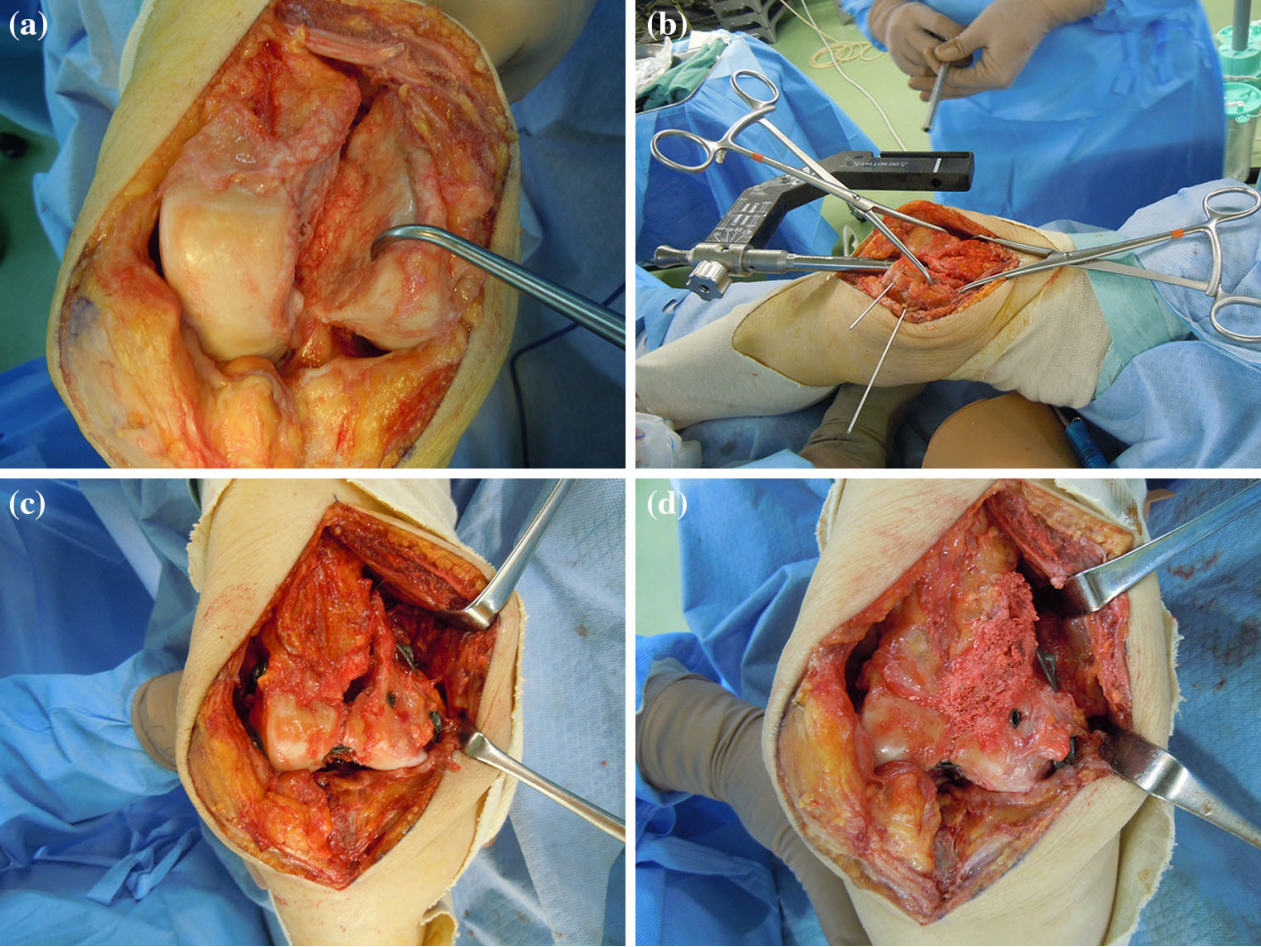
**a** Photograph depicting the inside of each sclerotic bone fragment after the resection of fibrous tissue. **b** Reduction maintained by provisional fixation and insertion of the nail. **c** Bony defect evident between the fragments. **d** Bone grafting to repair the defect

**Fig. 3:**
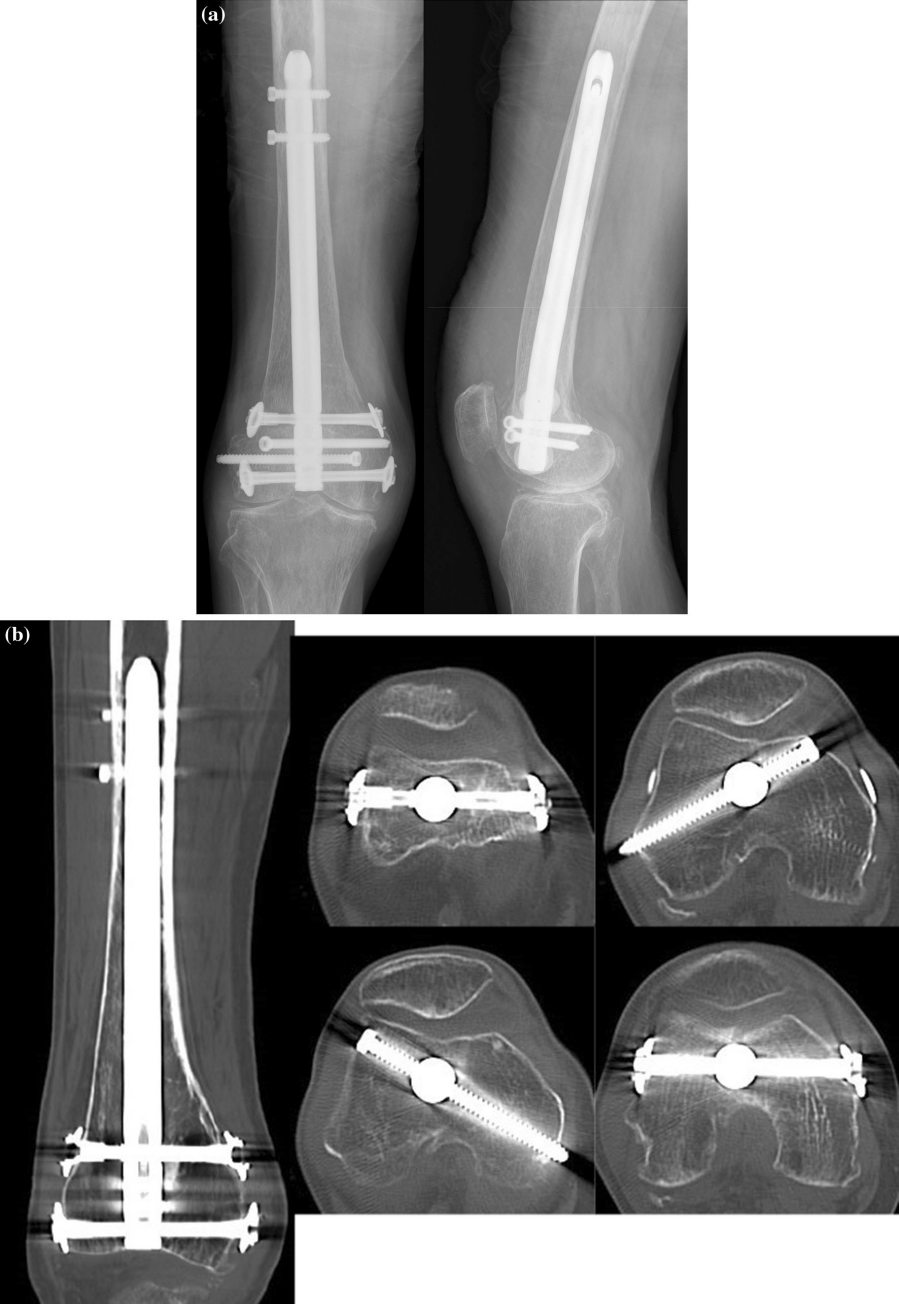
**a** Radiographs demonstrating bony union and no loss of reduction. **b** Computed tomography demonstrating bony union and suitable congruity

Surgery was performed to obtain bony union and regain knee joint stability. The knee joint was exposed by a medial parapatellar approach. Fibrous tissue had formed between the bone fragments and was removed completely. The inside of each bone fragment appeared sclerotic and the distal condyle of the femur appeared as two distinct bones (Fig. 2a). The sclerotic bone inside each bone fragment was refreshed and bleeding from the refreshed bone surfaces confirmed. The displacement was reduced directly, and the reduction maintained provisionally using Kirschner wires and forceps (Fig. 2b). An anatomical reduction was achieved. The entry point for the retrograde nail was identified at the center of intercondylar notch as routine in the method. The entry point was perforated carefully and reaming was performed only for the condyle and metaphyseal area. A T2 supracondylar nail (Stryker, Tokyo, Japan) was inserted (Fig. 2b). A 200-mm nail of diameter 13 mm was used and was of sufficient length to reach the femoral isthmus (Fig. 3a). The depth of insertion was adjusted in order to insert four locking screws into the femoral condyle. Two 5.0-mm fully threaded locking screws and two 5.0-mm “condyle screw and condyle nut” pairs were inserted into the condyle. An end cap was inserted onto the distal end of the nail and the most distal condyle screw locked. Two 5.0-mm fully threaded locking screws were inserted proximally into the shaft. A bone defect was still evident at the anterior aspect and within the nonunion site (Fig. 2c); bone grafting, in the form of a combination of autologous bone harvested from iliac crest and beta-tricalcium phosphate, was used to fill the defect (Fig. 2d).

The postoperative course was uneventful. Partial weight bearing was allowed from 6 weeks postoperatively and full weight bearing from 12 weeks. Bony union was successfully obtained (Fig. 3), and the previous gross instability eliminated. The patient regained the ability to walk unaided by 3 months postoperatively. The range of motion of the knee was 0°–115°. At the last follow-up, 16 months after the surgery, no loss of reduction or implant failures was observed, and the recovered function maintained.

## Discussion

Articular fractures may develop into a pseudoarthrosis if accurate fracture reduction (in order to maximize joint congruity and fracture surface contact) is not accomplished together with stable fixation. This case is an instructive case demonstrating that inappropriate management out with established principles for articular fractures leads to development of a pseudoarthrosis.

This patient is elderly and presented with accompanying comorbidities: diabetes and osteoporosis. Despite the accompanying medical conditions, the patient had been fully mobile prior to the injury and was sufficiently well for surgery to be considered. Consideration was given to screw and plate fixation but excluded as standard anatomical locking plates for distal femur are applied onto the lateral condyle. Whilst there are plates designed for medial application, these are in the context of fixation of distal femur osteotomy and not for fracture fixation. A decision was taken to use an intramedullary nail with secure fixation of the medial condyle provided by pairs of condyle screw and nuts which are implant accessories designed for use in osteoporotic bone. We considered that suitable compression between the fragments could be applied and congruity maintained using this system. On the other hand, we identified that this patient also had an accompanying bony defect between the fragments, and we judged that achieving suitable levels of angular and rotational stability only using the “condyle screw and condyle nut” system would be insufficient. Therefore, we planned the surgery as follows: apply suitable compression to the fracture using the “condyle screw and condyle nut” system, achieve the angular and rotational stability by adding an intramedullary nail, and improve stability through bone grafting at the defect site. We chose the T2 supracondylar nail for this procedure, as it has a multiple, multi-directional distal locking system. The nail was inserted passing the isthmus to obtain the stability of the nail/screw construct. We controlled the depth of nail insertion and inserted a maximum of four screws including two units of the “condyle screw and condyle nut” to apply and maintain suitable compression between the fragments. The most distal screw was locked to the nail hole by the end cap, and this contributed the improvement in angular and rotational stability.

Wähnert et al. [6, 7] performed a biomechanical study which compared four fixation devices in an osteoporotic synthetic bone model of an AO/OTA type 33-C2 fracture. One angular stable plate with three intramedullary nails, differing in their mechanisms of distal locking [with two lateral-to-medial screws in one construct, one screw and one spiral blade in another construct, and four screws (two oblique and two lateral-to-medial with medial nuts) in the third], was investigated biomechanically under torsional and axial loading. Two nail constructs (the one-screw and spiral blade construct and the four-screw construct) were also compared under axial loading in fresh-frozen human cadaveric femora. Their conclusions were as follows: (1) For intramedullary nails, the kind of distal interlocking pattern affects the stabilization of distal femoral fractures; (2) four-screw distal locking provided the highest axial stability and nearly comparable torsional stability to that of the angular stable plate; and (3) the four-screw distal interlocking construct was found to have the best combined (torsional and axial) biomechanical stability. In their study, the T2 supracondylar nail with the “condyle screw and condyle nut”, as used in our surgery, was used as the intramedullary nail with four-screw distal locking. Although the fracture type of our case (type B2) was different from the one in their study (type C2), this biomechanical test provided us with useful information. Their type C2 fracture model consisted of an intercondylar fracture and an additional fracture gap at the supracondylar area. Therefore, the fracture model represented a more complex fracture pattern, as compared with a type B1 or B2 fracture. It was suggested that if torsional stability was required (e.g., for bedridden patients), an angular stable plate will be sufficient; but, in contrast, a supracondylar nail should be considered for mobile patients where early postoperative mobilization for rehabilitation was desired. This patient was previously mobile, and early postoperative mobilization for rehabilitation was desired; this led to the choice of the supracondylar nail in implant selection. Although the fracture type (OTA 33-A3) differs from a unicondylar fracture, a more recent biomechanical study using fresh-frozen human femora revealed that the use of a washer/condyle nut as part of a distally locked IM nail provided superior fixation as compared to the unlocked version for distal fixation when using a retrograde intramedullary nail [8]. This adds support to the use of the “condyle screw and condyle nut” in locked fixation to improve stability.

## Conclusion

Plate and screw fixation is an effective choice for young patients, but for elderly mobile patients with osteoporosis, a supracondylar nail with the “condyle screw and condyle nut” fixation offers an alternative and as effective treatment option. If plate and screw fixation is applied to elderly patients, an angle stable system of locking plates should be considered. We reported a case of medial femoral condyle pseudoarthrosis successfully treated using retrograde intramedullary nailing with an enhanced form of distal locking. We believe this surgical method can be a useful alternative to angle stable plate options in treating such pseudoarthroses.
